# *Stachel*-mediated activation of adhesion G protein-coupled receptors: insights from cryo-EM studies

**DOI:** 10.1038/s41392-022-01083-y

**Published:** 2022-07-09

**Authors:** Ines Liebscher, Torsten Schöneberg, Doreen Thor

**Affiliations:** Rudolf Schönheimer Institute of Biochemistry, Medical Faculty, Leipzig University, 04103 Leipzig, Germany

**Keywords:** Structural biology, Structural biology

Recently, a set of cryo-electron microscopy (cryo-EM) structures of different adhesion G protein-coupled receptors (aGPCRs) has been published by Xiao et al.^1^, Ping et al.^2^, Qu et al.^3^, and Barros-Álvarez et al.^4^ shedding light on the activation of the seven-helix transmembrane domain (7TMD) via their tethered peptide agonist.

Adhesion GPCRs are an evolutionary old class of receptors that play a key role in several physiological processes including neuron and synapse formation, immune response, and metabolism. They are unique within the superfamily of GPCRs because they combine the structural features of adhesive molecules, mediating cell–cell or cell–matrix interaction, with intracellular G protein-mediated signalling. The long extracellular N terminus of the receptor harbours distinct functional domains including the highly conserved GPCR autoproteolysis-inducing (GAIN) domain with the GPCR proteolysis site (GPS). Many, but not all aGPCRs are autoproteolytically cleaved at the GPS into a N- and a C-terminal fragment (NTF and CTF, respectively), which remain non-covalently attached.

**Fig. 1:**
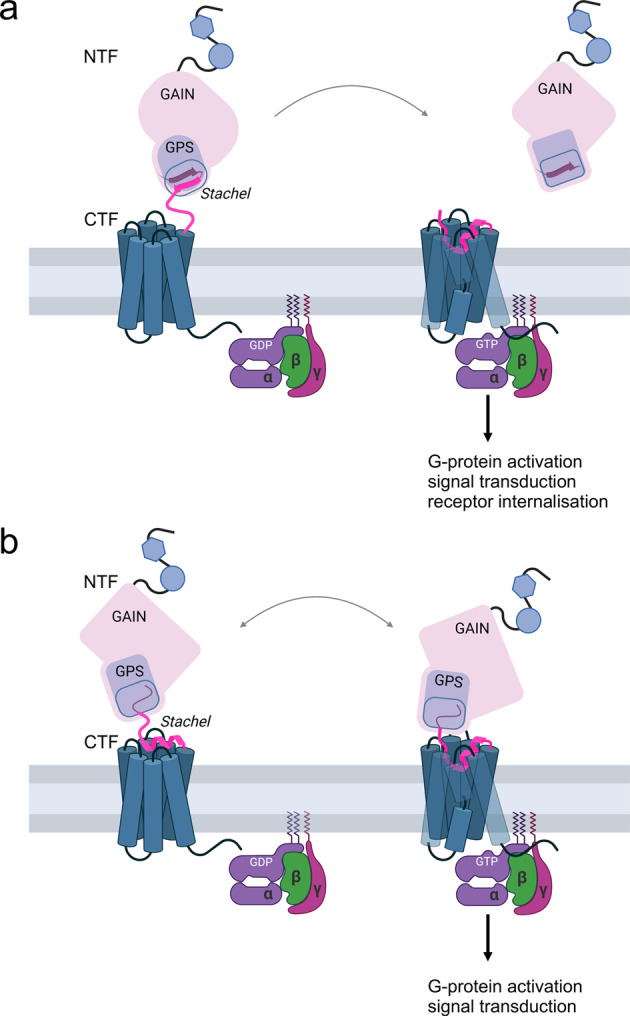
Potential activation of G-protein signalling of aGPCRs by their tethered agonist. **a** Irreversible dissociation model: In the inactive stage, the *Stachel* sequence is located as a β-sheet (magenta) within the GAIN domain. Following an adequate signal (e.g., mechanical force or ligand binding) the N-terminal fragment (NTF) is removed. The tethered agonist is exposed and binds to the 7TMD in the form of an α-helix resulting in G-protein activation. Because the NTF dissociates from the CTF, the receptor needs to be internalised and degraded for inactivation and cannot undergo another activation cycle (not shown). β arrestin-mediated as well as β arrestin-independent mechanisms have been described for aGPCR internalisation. **b** Reversible isomerisation model: In the inactive stage, the *Stachel* sequence is prebound as an α-helix to the 7TMD in its inactive or partially active conformation. Following receptor activation, the *Stachel* sequence isomerises into the fully active conformation resulting in G protein-mediated signalling. Because NTF and CTF remain attached, multiple activation cycles are possible and receptor internalisation is not mandatory, but might eventually also occur after receptor activation. The figure was created with BioRender.com

It was first demonstrated only a decade ago that the CTF displays higher basal activity compared to that of full-length receptors, and that deletion of the distal N-terminal amino acids of the CTF abolishes this constitutive activity. As a first step towards understanding the activation mechanism of aGPCRs, a tethered agonistic sequence within the N terminus of the CTF was identified. It was subsequently determined that several aGPCRs could be activated by synthetic peptides derived from this so-called *Stachel* sequence. Until recently, it remained unclear how exactly the tethered agonist is exposed to enable interaction with the 7TMD, a necessary step for the activation of G-protein signalling. Crystal structures of the isolated GAIN domain revealed the *Stachel* sequence as a β-sheet buried deeply within a twisted β-sandwich.^5^ Therefore, it was concluded that the NTF needs to be removed to expose the *Stachel* sequence and achieve receptor activation (Fig. 1a). Different mechanisms were proposed to initiate the dissociation of the NTF from the CTF following auto-cleavage including interaction with extracellular binding partners and mechanical forces. However, autoproteolysis does not occur in all aGPCRs; therefore, their activation cannot be explained by the exposure of the *Stachel* sequence due to NTF removal.

Now, the published cryo-EM structures of CTFs for different aGPCRs provide molecular insights into the arrangement of the activated receptor and its built-in peptide agonist together with the interacting G protein. The studies identified a hydrophobic motif that is conserved within the *Stachel* sequences and is crucial for the hydrophobic interactions with the 7TMD binding pocket.^1–4^ In contrast to the ß-sheet fold of the *Stachel* sequence within the GAIN domain, the hydrophobic motif of the tethered peptide agonist bound to the 7TMD shows an α-helical structure (Fig. 1). A similar binding mode was found for a *Stachel* sequence-derived peptide.^1^ These CTF structures can now be used to develop agonistic or antagonistic peptides specific for certain aGPCRs. Most interestingly, the cryo-EM structure of a cleavage-deficient full-length construct of ADGRF1/GPR110 showed the *Stachel* sequence already positioned at the 7TMD binding pocket challenging the current model of NTF removal for receptor activation.^3^ Indeed, several studies have shown that GPS cleavage is not required for aGPCR function in vitro and in vivo. Furthermore, the new cryo-EM structures of aGPCRs provide characterisations and comparisons of the 7TMD-G protein contact interfaces, thereby offering molecular insights into G-protein selectivity.

Taken together, these new cryo-EM structures shape our models of aGPCR activation, which can be now summarized in two different scenarios (Fig. 1):

(i) In the inactive state, the tethered peptide is buried as a β-sheet within the GAIN domain. Following autoproteolytic cleavage and removal of the NTF (e.g., by mechanical forces), the agonistic *Stachel* sequence is exposed and can interact with the 7TMD, where it adopts the shape of an α-helix. For inactivation, the receptor needs to be internalised and degraded (irreversible dissociation model) (Fig. 1a).
(ii) In the inactive state, the tethered peptide is prebound to the 7TMD already in the form of an α-helix. Mechanical forces or interaction with allosteric ligands result in conformational changes of the large N terminus, which are transduced into an isomerisation of the *Stachel* sequence and subsequently lead to receptor activation. In this scenario, removal of the NTF is not necessary, and the receptor is able to undergo multiple activation cycles (reversible isomerisation model) (Fig. 1b).

In support of the first hypothesis, mutations in the GAIN domain were identified that reduced the interactions with the tethered peptide sequence and an increase in basal activity indicated a release of the tethered peptide from the GAIN domain.^1^ However, the same effect was observed in a cleavage-deficient mutant,^2^ indicating that these mutations promote the activation state even without NTF removal. Support for the second hypothesis comes from a cryo-EM structure solved by Qu et al.^3^ Although the NTF of full-length structures is not solved most likely due to high flexibility, analysis of a cleavage-deficient full-length ADGRF1/GPR110 showed the *Stachel* sequence already in the 7TMD binding pocket as found in the CTF structures.^3^ Such a prebound tethered agonist isomerising upon extracellular ligand binding was identified as the activation mechanism for another group of GPCRs with large N termini - the glycoprotein hormone receptors. To decide which of these two models – or perhaps both – represents the actual mode of activation will require high-resolution structures of full-length aGPCRs in their inactive and active stages.
